# Acceptability, feasibility, and ethics of saliva collection in community-based research with Mexican-origin mixed-status families during high immigration enforcement

**DOI:** 10.1186/s12889-022-13903-5

**Published:** 2022-09-05

**Authors:** Airín Denise Martínez, Lillian Ruelas-Thompson

**Affiliations:** 1 School of Public Health and Health Sciences, Department of Health Promotion and Policy, University of Massachusetts-Amherst, 715 N. Pleasant Street, Arnold House 333, 01003 Amherst, MB USA; 2 College of Liberal Arts & Sciences, ASU Advance,, Knowledge Exchange for Resilience, Arizona State University, Box 5302, Tempe, AZ 85281 USA

**Keywords:** Biobehavioral research, Community-based research, Immigrants, Immigration enforcement, Latinx, Mexican-origin, Salivary biospecimens

## Abstract

**Background:**

There are concerns about the representation of vulnerable and underrepresented racial-ethnic minorities in biomedical and public health research, particularly when the research requires the collection of biospecimens. The current paper reports on the acceptability, feasibility, and ethics of saliva collection in a study examining the relationship between chronic stressors among mostly mixed-status, Latinx families (*N* = 30) during high immigration enforcement.

**Methods:**

Data for this study included anthropometric measures and salivary biospecimens from each family member (*N* = 110) and a household survey. Data for this analysis are from ethnographic field notes, which were analyzed using a bricolage of critical ethnography and case study analysis techniques.

**Results:**

We discuss the feasibility, aversions, acceptability, and ethical implications of integrating salivary biomarkers with Mexican-origin mixed-status families living in an area with restrictive immigration enforcement policies. We present the recruitment and data collection strategies used by the research team to gain participants’ trust, retain families, and maintain confidentiality.

**Conclusion:**

We recommend that researchers who obtain biospecimens from Latinx, Mexican-origin, and/or immigrant populations answer the participants’ questions honestly and without fear that they will not understand the science to obtain voluntary assent and consent. We recommend that researchers be knowledgeable of the sociopolitical context that the Latinx, immigrant, and in particular, mixed-status families inhabit so that they are prepared to provide informational resources. Finally, we think it is imperative that the study team in the field be bilingual, multicultural Latinx persons who identify with the community.

## Background

There are concerns regarding the representation of vulnerable and underrepresented racial and ethnic minorities in biomedical, clinical, and public health research. Recruiting and retaining diverse populations for research is important if we are to develop treatments and community-based and structural interventions that reduce health inequities. Recruiting and retaining diverse populations is also important given a history of racism in medicine and public health that has produced racial inequities in screening, disease risk factors, and treatment effects [1–3]. Despite the passage of the National Institutes of Health (NIH) Revitalization Act of 1993, which mandated the inclusion of women and racial-ethnic minorities in NIH-funded research, minority populations remain largely underrepresented in U.S. health research [4]. While Hispanics/Latinx represent 18% of the U.S. population, since 1993, less than 4.4% of the NIH research program grants have focused on the Hispanic/Latinx population [5]. Despite this low representation, minority adults report being willing to participate in health research [6, 7]. Participation of Hispanics/Latinx persons (from now on *Latinx*) in biomedical, clinical and health research is not representative of their numbers in the U.S. population as the largest racial-ethnic group in the United States (> 60 million) [8].

Some of the reasons why there is much lower participation of Latinx persons in biomedical and public health research can be attributed to “past atrocities in medical experimentation, cultural differences in health beliefs and practices, power imbalance [between health researchers/providers and the participants/patients], communication challenges, and issues related to health system organization” [9]. In addition, participation in biomedical and public health research competes with Latinx persons’ time for work and family caregiving [10]. Latinx persons are also concerned about adverse reactions and infections from clinical trial treatments [11] or the stigma related to learning of one’s disease status (e.g., HIV status), [12, 13] not to mention the lack of health insurance coverage to treat discovered disease [9, 11]. There is also the fear of immigration enforcement (e.g., detention, deportation and family separation) [10, 14–16]. Important to note is the lack of multicultural, bilingual Master’s- and doctoral-prepared biomedical and health researchers who can readily relate and communicate with Spanish-speaking and indigenous language populations of the Americas [4]. Latinx persons may also be less likely to participate in health research if it requires biological specimens (e.g., blood sample, genetic sample) or participants have to use invasive medical equipment [7].

There have been requests to examine how racial and ethnic minorities embody discrimination and systemic inequalities throughout the life course [17–19]. However, there has been little research examining how Latinx persons embody racial and ethnic discrimination, much less how structural racism from policies and institutional practices affect physiological mechanisms related to chronic disease. Many interdisciplinary health researchers are turning to salivary analytes to measure biomarkers representing acute and chronic stress vis-à-vis the hypothalamic-adrenal-pituatary axis, endocrinological processes, and local and systemic inflammation. Salivary biomarkers are growing in popularity because they are less invasive than venipuncture, do not require fasting before collection of the sample, and are easy to store for later analysis [20]. Children may also be more cooperative providing a saliva sample than a blood sample [21].

The current paper reports on the acceptability, feasibility, and ethics of saliva collection in a community-based study examining the relationship between chronic stressors, including fear of immigration enforcement and perceived racism, among mixed-status Latinx families in Phoenix, AZ. Mixed-status families refer to families with one or more immigrant family members who do not have legal authorization to live or work in the country they reside. Latinx persons compared to Non-Latinx Whites suffer disproportionately from cardiometabolic risk with the highest prevalence of female adult [22] and pediatric obesity, [23] youth metabolic syndrome [24, 25], and prediabetes [26] in the United States. Latinx persons that are more vulnerable to health inequities are those: 1) with darker skin, 2) whose primary language is not English, 3) without authorized immigrant status, and/or 4) persons belonging to a mixed-status family. For example, persons with unauthorized immigrant status are vulnerable in immigrant-receiving countries like the United States because there has been an increase of immigration enforcement policies and practices after the terrorist attacks of 9/11 [27].

An estimated 16.7 million U.S. citizens live in a household with at least one unauthorized immigrant, or a mixed-status family [28, 29]. Unauthorized immigrants in Arizona, where the present study takes place, are restricted from obtaining state-issued identification, including driver’s licenses, participating in public health insurance and poverty-reduction welfare programs [30, 31]. Immigration enforcement adversely affects U.S. citizens as well because those with unauthorized family members are excluded from gainful employment, enrolling in public health insurance programs, and in many states, omitted from household calculations for welfare programs like Supplemental Nutrition Assistance Program [28, 32]. Moreover, there is always the threat that an unauthorized family member, particularly parents, being apprehended, detained, or deported, causing family separation [31, 33]. It is estimated that six million minor children in the United States are in a mixed-status home [28]. These conditions produce collective fear and stress in mixed-status families and among Latinx communities, whom are often targeted in these policies [34]. Unauthorized and authorized immigrants as well as members of mixed-status families should be considered “vulnerable and in need of protection” [14].

Brabeck and colleagues [35] assert that researchers face major ethical challenges working with unauthorized immigrants because researchers are limited in the help they can provide their participants, as we cannot change the immigration and social welfare policies that exclude them and their families. Moreover, they indicate that research findings have the potential to further ostracize migrant communities (e.g., reporting information that could harm them) and produce more than minimal risk, if our participants’ data falls into the hands of local law or immigration enforcement. Despite unauthorized immigrants’ vulnerability, we should approach unauthorized immigrants and their family members as simultaneously capable and competent to avoid further marginalizing and disempowering them in the research encounter [14, 33].

Our unique contribution to the literature is that we present the feasibility and ethical implications of integrating salivary biomarkers in Mexican-origin, mixed-status families. Although other researchers [32, 36] demonstrate the feasibility of collecting salivary biospecimens from Latinx migrant farmworker populations, they do not distinguish experiences between authorized and unauthorized immigrants and those persons in mixed-status families living in an urban area with high immigration enforcement. Nor do these researchers discuss the potential challenges for research participants to collect additional saliva samples throughout the day, independent of the research team. We hope to provide health researchers with tangible tools and recommendations for recruiting and ethically attaining the participation of Latinx persons, particularly those experiencing legal vulnerability from the criminal justice or immigration enforcement systems, for research that collects anthropometric and salivary data.

## Methods

### Positionality statement

We acknowledge that researchers’ positionality shapes the research situation with the participants. This research was conducted by two Latinx women, one graduate research assistant at the time (LRT), and one academic (ADM). Each of us has a distinct life trajectory and perspective because we work for the university in different capacities and have different educational and cultural backgrounds. LRT is a Mexican American, transborder Sonoran resident. LRT is fully bilingual but is white-passing because she has fair skin and blue eyes. LRT’s family is from Sonora, and she often commutes between Phoenix, Tucson, AZ, and other Sonoran cities to visit her family in Mexico. Her knowledge about Mexican transborder communities was vital for ADM to learn about Arizona’s Latinx and Mexican communities. For example, many people operate businesses from apartment living rooms selling prepared foods, sundries, and textiles because they often live in resource-poor areas. LRT also alerted ADM to gendered, outdoor activities on the weekend such as grilling, cleaning and church.

ADM acknowledges her power and privilege as a middle-class, academic researcher to represent other people’s stories and experiences. She shares a racial-ethnic identity and former class position as a multiracial (not white-passing), Latinx woman from a working-class, Puerto Rican and Guatemalan mixed-status family in Chicago. However, the major wall that keeps her from being a representative of the Phoenix Latinx community is that she is not from Arizona, she is not Mexican, and was affiliated with a university that had contentious relationships with some Arizona communities. Some community members we tried to recruit into this study brought to our attention that some university researchers have conducted opportunistic research and did not remain committed to their community partnerships or sustained interventions.

In relation to the immigration enforcement environment in the United States, both LRT and ADM have family members who were and/or are unauthorized immigrants living in the United States. We understand mixed-status families avoid state and public agencies, even when they need them, to protect unauthorized family members from discovery and potential removal. We can only imagine the fear that unauthorized people have moving in public space. For example, in 2015, while walking in her Downtown Phoenix neighborhood to run errands, ADM was stopped by a police officer on foot and asked to show identification. Although she could produce identification, not having those documents at that moment with police can be the difference between being free and being in a local jail or immigration detention facility until one’s identity and immigration status are verified. We understood that the stakes were very high for mixed-status family members to unintentionally disclose whether they, or someone that they live with, are unauthorized migrants. We entered this project agreeing with many scholars that exclusionary immigration enforcement policies are forms of institutional racism that have consequences for families’ social, emotional, and financial wellbeing [15, 17]. We sought to demonstrate the physiological consequences of these policies and practices on parents and their children.

In addition to Latinx communities and the immigration enforcement environment in Arizona, we acknowledge our position relative to this being our first experience conducting biobehavioral research integrating biospecimens. Although both authors have previous experience *living* and *working with* Latinx communities, as well as conducting participatory quantitative and qualitative research with these communities, we never requested consent to collect biospecimens from Latinx persons. We believed these procedures could be interpreted as intrusive of the participants’ embodied privacy. Admittedly, it was initially uncomfortable for us to ask for so much data from families because they received so little in return, except a small monetary incentive.

Our positions within academia and in the community are both contradictory and tenuous. Despite how many identities and experiences we may have shared with our participants, we cannot automatically speak on their behalf as low-income, unauthorized immigrants, and for many English is not their first language. Nevertheless, our positionality informs the aversions, acceptability, feasibility, and ethics that we identified in conducting biobehavioral research with mostly mixed-status Latinx families.

### Bricolage of critical ethnography and case study approach

This analysis utilizes a *bricolage* [37] of critical ethnography [38] and multiple case study approach [39] to explore how Latinx families along the Southwestern borderlands, could affect their desire or hesitation to participate in a study collecting saliva specimens. The multiple case study approach was used to capture experiences of multiple families and to identify insights about the research procedures and saliva collection from the children, youth, and adults. We integrate elements of critical ethnography to determine the acceptability, feasibility, and ethics of collecting biospecimens from a historically marginalized group: Mexican-origin persons in the context of a state with high immigration enforcement. We integrate elements of critical ethnography because the initial intent of this study was to examine how inequities resulting from being and/or living with an unauthorized immigrant in a social environment hostile towards Latinx populations and immigrants is related to physiological proxies for stress and inflammation, or how immigrant illegality and its spillover effects are embodied in families. Given prior research that indicates that mixed-status families have adverse cognitive, education and self-rated health outcomes, we expected most families we approached to mistrust our intentions and decline participation.

### Recruitment

Data for this paper are drawn from the researchers’ experience conducting a community-based biobehavioral study in Phoenix, Arizona. The primary goal of the study was to distinguish how diverse chronic stressors, including immigration, family conflict, fear from immigration enforcement, marital and parental chronic stress are related to salivary biomarkers for stress (e.g., alpha amylase, cortisol, uric acid) and inflammation (pro-inflammatory cytokines) in Latinx families, with at least one immigrant parent. A secondary goal was to assess the feasibility, acceptability, and ethics of collecting salivary specimens and anthropometric measures in state with heightened immigration enforcement, specifically the implementation of Arizona Senate Bill 1070 (for more on SB 1070, refer to Magaña & Lee) [40].

The lead author has previously dealt with issues of mistrust between the participants and her research team by recruiting participants through collaborations with community-based organizations (CBOs) [41, 42]. However, there were times when participants made her aware that they had experienced class and immigrant status discrimination from bilingual service providers at a partnering CBO. There was also criticism from academics that using a convenience sample produces selection bias—mostly low-income, Latinx women with children seek assistance from CBOs, not representing the general population.

Therefore, for this study we recruited families using a clustered probability sampling strategy. We conducted a simple random sample of census tracts with a large proportion of foreign-born Hispanic/Latinx persons in Phoenix, and then a random selection of block groups with a high proportion of Hispanic/Latinx persons. The team then went door-to-door describing the study (in the person’s language of choice) and finding families with at least one Latinx immigrant parent and one child living at home. We disqualified families from participation if the head of household was incapable of providing consent for themselves or their children. For the validity of the salivary analytes, following recommendations by Granger and colleagues [43], we excluded families who had a family member that: just visited the dentist in the last 24 h; smoked or chewed tobacco; had open mouth sores or abrasions; ill with an acute condition or chronic disease; or a had a fever. We excluded families that had a person that was ill with an acute or chronic cardiometabolic condition because our pilot study examined proinflammatory cytokines. Proinflammatory cytokines become elevated in the presence of injury, illness, and infection. Although we sought diverse Latinx subgroups, given the demographic composition of Phoenix, all our families were Mexican origin. One out of every 13 families we spoke to in the field qualified to participate in the study. However, most families could not participate because they had at least one family member with a pre-existing chronic disease.

### Analysis

Data for this analysis are from participant observation field notes from both authors about our visits with each family, their demographic responses to the household survey to describe the sample, and the participants’ physical artifact: their saliva specimen. We met with each family at least three times: 1) the first to describe the study and schedule a time when all family members would be available to participate in the study, 2) the second to obtain consent and assent and collect data from each family member, and 3) the third to retrieve additional saliva samples and clarify any remaining questions. The time between visits was usually between two to six days. Observations of our interactions with the participating families were completed by both authors.

Immediately upon returning from the field, one author would draft notes about the experiences recruiting and collecting data that day. The field notes had a structure that stated the conditions for recruitment, a description of the family, our interactions with the family, and challenges with the whole process. Within 24 h, the other author would immediately review the draft and add their perspective or certain details that they found important to document. The field notes would also document more human moments such as children’s fascination with our equipment, families’ questions about the research, the saliva collection experience, and any information outside of the research questions that the families volunteered to share with us. Lastly, we documented the head of household’s recollection of their experience providing saliva throughout the day, independent of the research team. We asked the head of household to collect four additional samples throughout one day for us to produce a diurnal cortisol curve. Each family had their own data file for later textual coding in *Atlas.ti 8.1.*

Moreover, we are examining the interpretations of public health research and biospecimen collection among a historically marginalized racial-ethnic group. We integrate analytic elements of case study to provide a description of patterns about our interpretation of the research participants’ experiences and beliefs about providing saliva in the context of high immigration enforcement and living in a mixed-status families. Multiple cases were selected to show the aversions, challenges, and transgressions (both positive and negative) to the research protocol. We wrote our field notes not only to capture family’s reactions to providing saliva for this project, but also to capture the way *we felt* asking adults, youth, and children for their time to answer a long survey, measure their waist and hip circumference, height, and weight, and donate saliva.

Both authors analyzed the data, which took place months after completing data collection with all families. We integrated inductive strategies [39] to identify patterns in the participants’ analysis to develop a thematic codebook, which was then used to mark text from our field notes. We wrote memos to describe individual themes, and subsequently, to discuss the relationships between the themes such as the processes and conditions that should be considered in future research to increase scientific rigor, but more importantly, the integrity and respect for Latinx, mixed-status families. Below we describe the saliva collection procedures to demonstrate the labor and potential inconveniences that participants experienced to provide saliva samples.

### Measures and procedures for the biobehavioral parent study

Instruments for the parent study included a household survey, collecting weight, height (or length for children < 2 years of age), waist and hip circumference, and a whole, unstimulated saliva sample of 1.5–1.8 ml (~ 1 teaspoon) from each family member at the same time. A detailed description of the psychometric measures in the survey can be found in another article [16]. The saliva was obtained using the passive drool technique from all adults and youth older than 5 years of age. Twenty-two children under the age of six sat on their parent's lap while the research team held a saliva child swab in their mouth for three minutes [43]. Samples were immediately stored and transported in a portable cooler containing dry ice. At the end of each day, participants’ de‐identified saliva samples were transported to the Institute for Interdisciplinary Salivary Bioscience Research (IISBR) where they were frozen at − 80 °C until the day of assay.

**Fig. 1:**
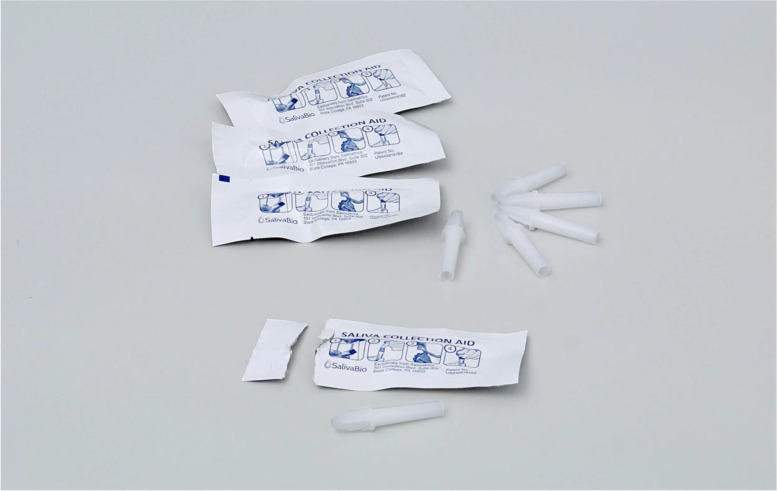
Saliva collection aids and cryovials. Source: https://www.salimetrics.com/device/saliva-collection-aid-sca# Image Courtesy of Salimetrics

**Fig. 2:**
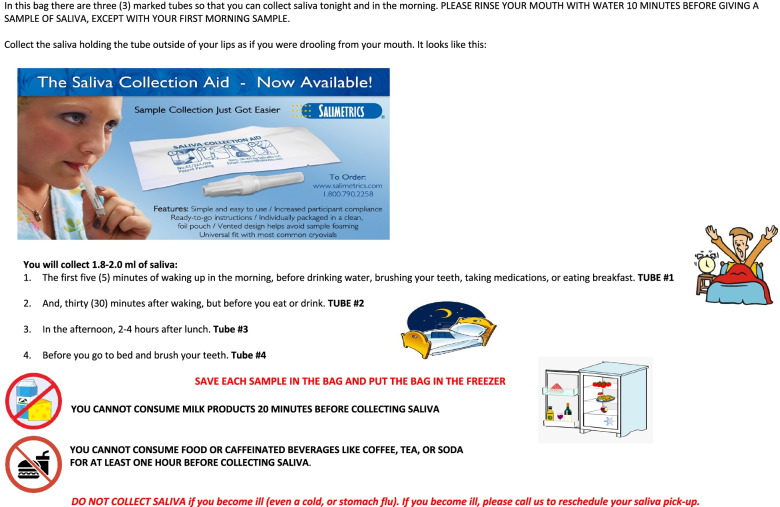
Instructions for Saliva Collection (created by authors 1 July 2014) Sources: Saliva Collection Aid image is courtesy of Salimetrics. The images of the bed, waking person, refrigerator, no dairy, and no food images are free for commercial use and no attribution is required. Available at: clipart-library.com

For the head of household to collect the additional saliva samples accurately and independent of the research team, we provided four saliva collection aids (See Fig. 1), four cryovials, a resealable plastic bag with the research team’s contact information and an instruction card in their language of choice (See Fig. 2). The research team reviewed the instruction card with each head of household. Each vial was marked with a permanent marker at the 0.5 ml line to indicate the minimum amount of sample needed per collection time. The instruction card indicated that the participant was to collect three additional samples: one within five minutes of waking, one 30 min after waking, one in the afternoon (2 to 4 hours after eating lunch), and one before bed.

Before leaving the participants’ homes, the research team obtained from each head of household their sleep and wake times to send a text message about 10 min before those times to remind the participant to collect a saliva sample. Those text messages also reminded participants not to brush their teeth, eat dairy or caffeine prior to providing sample. Water was allowed. After collecting a saliva sample, the participant was asked to seal their sample, return it to their resealable plastic bag, and place the bag in the refrigerator. We told the participants that if they fell ill within the 24 h of our home visit that they contact the research team to collect their saliva samples on another day when they were healthier. Fortunately, none of the heads of household became ill during the study.

## Results

### Participants

Our sample consisted of 30 families (*N* = 110). We had 46 adults (> 18 years of age), 12 children < 2 years of age (58.3% female), 15 children 3 to 5 years of age (46.7% female), 24 children 6 to 12 years of age (50% female), and 13 youth 13–18 years of age (38.5% female). The average family size was four persons with a range of two to eight persons per family. The number of children living at home ranged from one to six persons, with an average of two children per family. Most immigrant family members lived in the United States for about 10 years (See Table 1).

**Table 1:** Socio-demographic family characteristics

	**Families *N* = 30**
**Family Size** [Range, (Mean)]	2–8 persons (4.2)
**Number of Children**	1–6 children (2.3)
**Years in USA** [Mean ± SD]	10.69 ± 7.49
**Years in Phoenix**	9.91 ± 6.75
Race, Head of Household	
*White*	7 (23.3%)
*Moreno (Black, Hispanic-origin)*	5 (16.7%)
*Mestizo (Indian & White)*	11 (36.7%)
*Zambo (Black & Indian)*	7 (23.3%)
Marital Status	
*Married*	23
*Living w/ partner*	3
*Divorced/Separated*	4
Annual Family Income	
< *$20,000*	16 (53.5%)
*$20,000-$34,999*	11 (36.7%)
*$35,000-$49,999*	3 (10.0%)
Home Ownership	
*Rent*	28 (93.3%)
*Own*	2 (6.7%)
Spanish survey	26 (86.6%)

Nineteen of the families were considered mixed-status families with at least one member of the family being an unauthorized immigrant. Although immigrant status was not directly asked in the survey, the family members often told us that they, or someone in their household, was an unauthorized immigrant. We may have not been able to obtain IRB approval had we included a question on one’s authorization status. We could verify the participants’ disclosure with our survey question about each family member’s insurance status. Most of the families (11/30) identified as mestizo, or having at least one indigenous and one European ancestor. Most families (26/30) had at least two parents/caregivers. More than half of the families reported an annual household income of < $20,000/year (See Table 1). Below we discuss the aversions, acceptability, feasibility, and ethics of integrating salivary biomarkers with Mexican mixed-status families living in an area with restrictive immigration enforcement policies.

### Sources of hesitation and challenges recruiting families

The first source of hesitation shared by many was the participants trying to decipher whether the research team were vendors or religious missionaries. In many immigrant enclaves and ethnic neighborhoods, it is common to have religious missionaries, food and cosmetics vendors go door-to-door to gain followers or sell products. The research team could have been easily mistaken for solicitors when people peeked outside their doors or windows because of the items we carried. We dressed in plain clothes, hauling an office cart with our portable stadiometer, scale, and portfolio, while the lead author had a backpack carrying study documents and a portable cooler. However, for some who did open their door, upon learning that we were not selling them anything, they were receptive to learn more about our study. Many of the families were very interested in a study examining the relationship between stress and chronic diseases like Type II diabetes. There were four cases when families initially declined to participate but then later approached the research team and requested to participate because they learned through a neighbor that they had a positive experience in the study. More importantly, we were not forcing them to buy anything or engage in any religious or political action.

The second source of hesitation to participate in our study came from Latinx youth between the ages of 16–24. These youth were concerned that we would be able to identify whether they had consumed illicit substances, particularly, cannabis. They shared with us that many low-paying retail jobs were conducting drug screenings with saliva samples. In Arizona many publicly-funded programs screen for illicit drug use. For example, residents with previous drug convictions in Arizona must conduct monthly drug screenings to receive benefits from the Supplemental Nutrition Assistance Program and cash welfare benefits (SB 1620). In one example, a mother of four teenage boys told her children that the research team would be able to find out through the saliva sample if they were consuming illicit substances. Young people and their parents alike associated the collection of saliva specimens with drug screenings.

Another source of hesitation from three parents was whether we were going to obtain information about their DNA from their saliva samples. One household that we met during recruitment did not qualify to participate in the project but were the directors of a community-based organization advocating for low-wage workers and immigrants’ rights. They were curious if families were hesitant to participate because we were collecting their saliva. They informed the research team about a research ethics controversy between the Havasupai Native Americans and genetic researchers at the university. This was the first time that the research team was made aware of this unethical research partly because the lead author was new to the Southwestern United States and was not given this information when she inquired from her colleagues about their perceptions of the Latinx community’s research experiences.

In this scenario the Havasupai commissioned genetics researchers at ASU to discover underlying genetic explanations for the increased prevalence of Type II Diabetes in their population. However, instead of producing research exclusive to understanding the etiology of Type II diabetes in the Havasupai, a series of genetics screenings and research were conducted that examined their genealogy, their migratory patterns, the genetic causes of alcoholism, and mental health issues [44, 45]. The most disturbing fact is that the Havasupai’s blood samples were being used for research unrelated to diabetes for over a decade unbeknownst and without the formal consent of the Havasupai research participants [46]. Although most Latinx families in our sample perceived saliva as less invasive than the collection of blood samples, they still had legitimate concerns about our ability to assay for other biomarkers, especially their DNA.

The last source of hesitation and challenge recruiting families to participate in this study was the perceived and actual burden of saliva collection. Although parents gave permission for their children to participate in our study and the children provided verbal (ages: infant-5 years) or written assent (ages: 6–18 years), once the time came to measure the children and collect saliva, some children expressed fear and discomfort. We experienced the most resistance and emotional distress from three toddlers between the ages of 1.5 to 3 years of age. For example, there was a two-year old little girl who had a meltdown after we asked her to be weighed on our SECA scale. There was another little boy, just under two years old, who wept at the site of the cryovials and swabs. The research team could not understand this response given that the families appeared calm and the children saw their parents and siblings being weighed, measured, and providing saliva samples. The parents of these children rationalized their children’s behavior from negative experiences with healthcare providers, especially doctors and dentists.

Similarly, for some heads of household, they were hesitant to participate in the study because they were concerned about maintaining fidelity with the research protocol for collecting saliva samples independent of the research team. For some, it was difficult to establish a pattern of their wake and sleep times if they had a job with an erratic work schedule or a night shift. Others were concerned about not being able to drink coffee or brush their teeth the first 30 min of waking and storing their saliva in their family’s refrigerator with their food. Below we discuss the ways that the research team was able to overcome these hesitancies and aversions to participating in our study.

### Facilitators for increasing the acceptability and feasibility of saliva collection

One of the most important reasons why the research team was able to overcome families’ concerns about participating in our study, was our ability to communicate about the research not only in a language that was comfortable for this community, but because we were a female, multicultural and bilingual team. As described earlier, prior to integrating biospecimens into her research the lead author had over 10 years of experience conducting community-based participatory research with Latinx communities in three U.S. cities. The second author is a local *Sonorense,* born and raised in Tucson, and with binational family ties on the Mexican side of the border. So, the research team consciously and openly identifies with this community.

We tried not to objectify the Latinx immigrant and Mexican-origin community as the Other, as *we identify with that community.* More importantly, we believe we were perceived as members of the community. The participants called us by our first names and did not perceive us as authority figures, despite being researchers. For example, there were three instances that families did not qualify to participate in our study, but they greeted us in their home with water or coffee. Those experiences showed us their trust in us, and showed their neighbors that we were not a threat. Nevertheless, this is not to say that we did not have to work for that trust.

For example, we overcame the community’s hesitancy to open the door to us for fear that we were salespeople or religious visitors, by developing a recruitment script that communicated our university affiliation and study accessibly in Spanish and English. We emphasized the importance of understanding how different stressors are related to people’s chronic disease risk, and they expressed it was conducive to their family’s concerns. They were interested in understanding and expanding knowledge about how different forms of stress affected their physical health or were interested in reversing policies related to Latinx and immigrant status discrimination. We assumed participants were competent and autonomous individuals, even the children. For example, if families appeared hesitant to participate, we left them with a flyer and a business card to consider and call us if they had questions. We never forced them to participate. We also understood that their time was valuable, so we always agreed to return at a day and time that worked for them and their family.

### Ethics of salivary biobehavioral research

We obtained consent from the head of household first by describing the consent form to them in their preferred language (English/Spanish). We did not assume the parents’ literacy or their familiarity with the research process. We wanted them to voluntarily consent and provide permission for their minor children to participate in the study. When we were conducting the study, between 2014 and 2015, we were four years post-implementation of SB 1070, which is known as one of the most draconian state-level immigration enforcement policies in the United States [47, 48]. There was a culture of fear around exposing one’s unauthorized immigration status because government agencies and local law enforcement under this policy act as an extension of immigration authorities. The consequences could be termination from work, family separation, detention, or deportation. So, we purposely sought to protect the identity of our participants, particularly those who were or lived with unauthorized immigrants.

We protected our participants’ identities by asking the head of household to choose a fictitious name and surname to be used on the written consent forms. Children under six gave verbal assent in their preferred language. Children six years and older provided written assent in the language of their choice. Children and youth then chose a pseudonym to sign their assent. The research team added the fictitious surname assigned by the head of household to connect the family’s consent and assent forms. Adult and child participants enjoyed the opportunity to choose a pseudonym that reflected cartoons, superheroes, and celebrities. The research team used the pseudonyms to document our participants in the field notes, track our gift incentives, and save our participants’ contact information on our research mobile phone for later retrieval of additional saliva samples. Once the research team completed data collection with a family, they deleted their names from the research cell phone.

The research team overcame participants’ concerns about how we would use their saliva specimens by not only being honest, but by taking as much time as participants needed to describe the research consent and assent process. We stressed that we did not receive IRB approval (we referred to it as “university approval”) to assay their saliva for drugs or genetic information. We explained to them that for any research project that they participate in, the consent form must explain what their biospecimens will be used for. Our consent forms did not state either the identification of drugs or genetic information. We even highlighted the language in their consent form so that they would trust us. We also told them that the lab that we were partnering with did not have the assays to test for drugs and that DNA was not germane to our project. We also indicated to them that our consent form stated that after the analyses, we had to destroy their samples. We also told the families and their children how to exit from the study if at any time they wanted to withdraw (or any study for that matter). They had the right to call the study team or the IRB and request to have their data withdrawn from the analyses, and we are obligated to respect their wishes. It was important to provide families information about their rights in a research study for them to engage in future research.

Now, when parents wanted to learn if their adolescent children were using illicit substances, we told those parents that we did not have permission to examine their children’s saliva for those purposes. However, we told parents in private if they were concerned about their children using illicit drugs that they could call a behavioral health provider in the resource list that we provided them. We also told parents (away from the youth) that there were at-home drug kits available at pharmacies and large retailers. We also took advantage of that private moment to discourage parents from suggesting that our research specimens would be used in any way to incriminate their children. The consent form clearly stated that we would not share any of their anthropometric, survey, or saliva data with government or immigration enforcement authorities. However, we realized in this scenario that our future research needs to include language in the assent forms that indicates that we would not share the children and youth’s interview, survey, or biological results with their parents, unless they are in grave danger. We understood that people and youth are concerned about sharing their experiences of stress, racial discrimination, and living in a mixed-status household with any government authorities [47–51]. In this project we did not have a certificate of confidentiality, which is issued by the NIH to protect the research participants’ privacy by banning disclosure of identifiable, sensitive research information to anyone external to the research team. Given this limitation, during the beginning of the study, the primary author contacted the university’s General Counsel to ensure that we would have legal representation if our research records were subpoenaed from the local police, local sheriff’s office, or immigration enforcement. The General Counsel could protect the university, the research team, and the research participants’ biological and demographic data from being subject to legal scrutiny. However, we could not provide legal protection for the family’s immigration-related issues. As Brabeck and colleagues remind us, even research informed by social justice cannot address inequitable access to legal representation in the U.S. immigration system [52].

In addition, as Cacari-Stone and Avila point out, in the United States we openly accept racial and ethnic minorities and unauthorized migrants as subjects in biomedical and public health research, but they are often denied health insurance and limited access to medical care [9]. Our consent form explicitly stated that the research team does not consist of clinicians so we cannot diagnose or treat a participant’s medical condition based on their saliva specimen. Over 50% of the participants in our sample were uninsured. Although we provided a resource list with free clinics and federally qualified health centers (which cannot deny anyone medical care because they cannot afford it), we could not guarantee that participants would get the help they needed. We also could not guarantee the quality of care they would receive at these facilities. In the future, we think it would be ethical to partner with community-based and charity clinics to help ensure people receive access to care.

Another ethical issue that the research team tried to overcome was the burden of saliva collection for some of our participants, especially the emotional toddlers, by acknowledging their concerns, being flexible with the data collection, and providing additional supplies. For example, we provided a distraught child with science-based coloring sheets and crayons to distract them. Once they were calm, we would attempt to resume saliva collection from the child. However, when a child did not calm down within 10 min, we asked the family if we could return on another day to collect data from the family because we did not want to cause distress in the child, or affect the cortisol and a-amylase measures. We did not want the parents coercing the children to provide samples, and equally, wanted to respect that child’s autonomy and not cause undue harm. In addition, the research team decided to work one Saturday a month to accommodate families’ work and school schedules. This really helped us maintain our follow-up visits with families. If the head of household was concerned about storing their saliva samples in their family’s refrigerator, we provided them an additional resealable bag to provide a barrier between their samples and their food shelves. Upon the participant’s request we provided disinfecting wipes and disposable gloves so that they could feel more confident reducing the transmission of microbes.

As was discussed above, some heads of household were concerned about maintaining the fidelity of the procedures to provide diurnal samples. Upon completing the household survey, the lead author described the saliva collection procedures. She drew a rough diagram of the diurnal pattern of cortisol to emphasize the importance of collecting saliva at the pre-designated times before bed and after waking. Ultimately, the research team was flexible. If they were concerned that the provision of saliva samples would interfere with the parent’s job, we gave them the option of providing their diurnal sample on their day off later in the week. We also reminded them that we would send them a text message reminder to collect sample during their bedtime, upon waking, and 30-min post-waking.

### Salivary biobehavioral research among mixed-status families

We entered this project assuming most people would refuse to participate because they would be afraid of us sharing their personal information and other data with government agencies, local law enforcement, or immigration enforcement authorities. Only twice did SB 1070 come up in our research encounters. Our very first family told us during the fear of deportation questionnaire that they no longer feared being deported, but that the head of household was deported in 2005. Despite being a legal permanent resident, this woman was deported for not carrying her state-issued identification during a traffic stop. Also, with our second family, the head of household’s brother was apprehended by Customs and Border Protection the day before our data collection.

This is not to say that the topic of immigration enforcement never came up in relation to our study or would not be an important issue to consider whilst conducting research as we transition between xenophobic and more liberal presidential and gubernatorial administrations. Nevertheless, the topic of immigration enforcement entered our communication mostly in relation to securing material resources for these families. For example, living as an undocumented Mexican made it difficult to obtain health insurance, gainful employment, and the ability to move between state lines and across the border for family emergencies. In one family, the father was a Cuban naturalized U.S. citizen and his wife was an authorized immigrant through his status. Their youngest child was a natal U.S. citizen, but the eldest son (17 years old) from an earlier marriage was an undocumented youth. The mother was struggling to keep her son motivated to remain enrolled in high school and asked us for information about Deferred Action for Childhood Arrivals. Mixed-status families mostly expressed frustration, not fear from the current immigration enforcement practices in Arizona, and the United States, more broadly. Given that the research team comes from and has relationships with low-income, immigrant, and mixed-status Latinx families, we knew that we had to enter this research with information to provide our participants about agencies, programs, and initiatives that facilitate immigration processes, provide access to low-cost health care, low-cost courses for the General Education Diploma and vocational training, among other services. These families saw us as a bridge to information about resources and we made the effort to connect them to the organizations that could address their needs.

## Conclusion

### Recommendations for salivary biobehavioral research with Latinx immigrant and mixed-status families

In a community-based sample of Mexican-origin families, we found that a biobehavioral, community-based research project that integrates salivary biospecimens is acceptable for immigrant families, even mixed-status families, if the research team is representative of the population under study. If the research leads are persons who do not speak Spanish or an indigenous language, lack experience working and living among Latinx populations, then it would be in their best interest to work with a health disparities researcher that identifies as Hispanic/Latinx and has the research experience. It is often a requirement to include aboriginal and indigenous persons in research with First Nations people in Oceania, Canada and some first nations in the United States [37, 53, 54]. Moreover, according to the United Nations, research consent with indigenous and native populations must be prior to the initiation of research, free of coercion and maleficence, and informed consent must be given collectively, not simply at the individual level [54]. Unlike the shared geography and identity of members of indigenous groups, it is difficult to obtain collective consent of all Latinx or Mexican-origin persons, who are a multilingual, multiracial, and multinational ethnic group, especially living in a large metropolitan area like Phoenix. Nevertheless, we were examining families as a unit of analysis. Although obtaining consent and assent was a collective process, we also tried to honor children and youth’s self-determination and autonomy if they did not want to participate in the research study.

This project is logistically feasible if the research team commits the time (1.5 to 3 h) for data collection and is flexible with each family’s schedule and living circumstances. We also suggest door-to-door recruitment in geographic units (e.g., census tracts) that have a large proportion of the population under study. We recruited families in diverse living situations: trailer parks, apartment complexes, houses, and apartment buildings. Meeting potential participants in their homes gave them more authority to decline participation and prevents the inconvenience of spending time and money to travel to a research site away from their neighborhood. Collecting additional saliva specimens to produce diurnal curves of specific biomarkers and reducing measurement error is possible if the research team describes the procedures simply, provides written and visual instructions in the participants’ preferred language, and establishes text message or phone call reminders to the participants. Some of the feasibility and acceptability articles cited here are older than five years old [36] and do not take explicitly discuss the increased state- and local-level immigration enforcement in the United States and anti-Latinx policies during the 2010s. Certainly the collection of biospecimens can produce fear related to surveillance and identification of deviant and criminal behavior, but researchers need to clearly indicate during the consent and assent process that identifying this behavior is not the research goal.

This study augments an emerging area of research describing the relationship between chronic stress from institutional racism and physiological health disparities among Latinx adults and youth. Our study provides researchers with a description of the conditions that facilitated the collection of salivary biomarkers in this population, which is vulnerable on their basis of their socioeconomic status, their racial and ethnic minority position, and their immigration status during heightened restrictions towards unauthorized immigrants and their citizen family members [49–51]. This will benefit researchers and policy makers who want to increase the participation of Latinx persons in biomedical and public health research and ameliorate health inequities that produce adverse biobehavioral health consequences and chronic disease.

## Data Availability

The datasets generated and/or analyzed during the current study are not publicly available because most of the participating families had one or more unauthorized immigrants. Other data related to this article can be found here. De-identified field notes used in this manuscript are available from the corresponding author on reasonable request.

## Abbreviations

9/11: The Terrorist Attacks on the United States on September 11, 2001
AZ: Arizona
DNA: Deoxyribonucleic Acid
HIV: Human Immunodeficiency Virus
IISBR: Institute for Interdisciplinary Salivary Bioscience Research
IRB: Institutional Review Board
NIH: National Institutes of Health
SB: Senate Bill
U.S.: United States
